# Oral foreign body granuloma to soft tissue fillers. Report of two cases and review of the literature

**DOI:** 10.4317/jced.54191

**Published:** 2018-02-01

**Authors:** Paris Tamiolakis, Evangelia Piperi, Panagiotis Christopoulos, Alexandra Sklavounou-Andrikopoulou

**Affiliations:** 1 Postgraduate Student, Department of Oral Medicine and Oral Pathology, School of Dentistry, National and Kapodistrian University of Athens, Greece; 2 Assistant Professor, Department of Oral Medicine and Oral Pathology, School of Dentistry, National and Kapodistrian University of Athens, Greece; 3 Assistant Professor, Department of Oral and Maxillofacial Surgery, School of Dentistry, National and Kapodistrian University of Athens, Greece; 4 Professor, Head of Department of Oral Medicine and Oral Pathology, School of Dentistry, National and Kapodistrian University of Athens, Greece

## Abstract

The increasing demand for cosmetic procedures in the orofacial area nowadays, results in a growing number of complications attributable to soft tissue fillers, including the development of foreign body granuloma. The purpose of this study is to present two additional cases of oral foreign body granulomas caused by liquid silicone and hyaluronic acid respectively and review the pertinent literature regarding the demographics, the clinical appearance, the histopathology and the treatment of these lesions.

** Key words:**Oral foreign body granuloma, hyaluronic acid, silicone, soft tissue filler.

## Introduction

Cosmetic procedures are considered a modern “weapon” against aging, a normal process that has been treated as a disease through time, mostly in the Western civilization. Nowadays, in an attempt to prevent esthetic changes as a result of aging, the use of injectable soft tissue fillers (STFs) is increasingly observed (1,2).

STFs are easy to handle and cause minimal side effects (3), hence, they are used in a wide range of aesthetic procedures, ranging from cheek and chin augmentation, nose reshaping and lip enhancement to hand rejuvenation (4). Despite the increased demand, a soft tissue material that would ensure long term aesthetic results along with low complication rate and low cost is not yet available in the market (2). Additionally, while only a few STFs have been approved by the Food and Drug Administration (FDA) (5) in the United States of America, a number of non-approved fillers, such as liquid silicone, are still in use to date either by trained physicians or, by unlicensed practitioners (6,7).

According to their biodegradability, STFs are classified by FDA into: absorbable (collagen, hyaluronic acid, calcium hydroxyapatite and poly-L-lactic acid) and non-absorbable (Polymethylmethacrylate (PMMA)) (5). Another classification is based on their duration and include temporary (absorbable), permanent (non-absorbable) and semi – permanent fillers (8). In the latter category a combination of both absorbable and non-absorbable STFs is used, with the temporary component acting not only to produce an immediate effect but also as a carrier, until the fibrotic reaction induced by the permanent filler takes place (9). The choice of the appropriate soft tissue filler (STF) to be injected depends not only on the desired outcome and the area to be treated but on the preference and the experience of the physician as well (10).

Usually permanent fillers cause complications; however, both clinicians and pathologists are faced with a growing number of side effects that may arise after injection of any type of STF(9). The time of occurrence and the type of complication vary among different fillers(2).The combination of different types of fillers injected simultaneously in the same area does not seem to be related to increased risk of complications (11-13). However when they do occur, it is more likely to be more severe and chronic compared to the use of a single filler (2). STFs complications may be either immediate, of early onset or delayed (4,7,8) ( Table 1). Among the delayed STFs complications, foreign body granuloma (FBG) formation is the most common histologic pattern (4) with a clinical incidence between 0.02 – 2.8% (8).

**Table 1:**
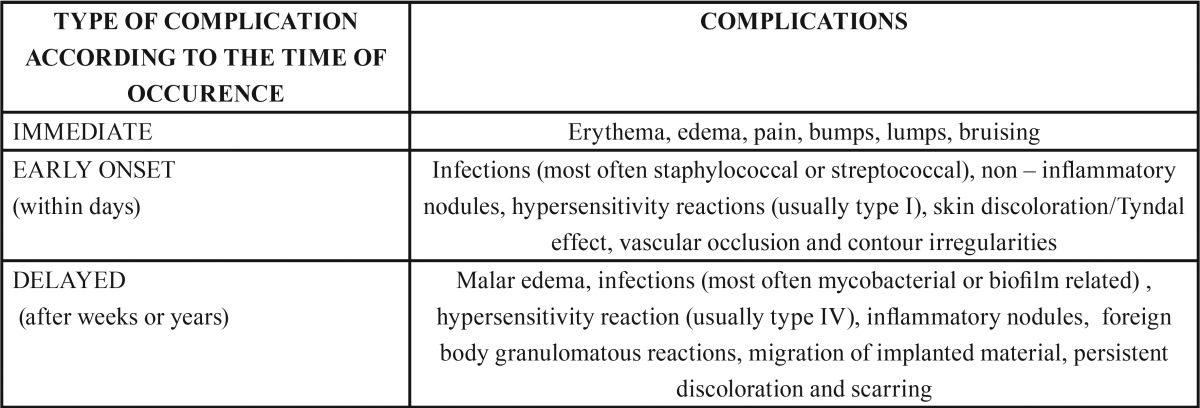
Possible complications after soft tissue fillers injections (4,7,8).

The purpose of this study is to present two cases of oral FBG to STFs and review the pertinent literature.

## Case Reports

Case 1

**Figure 1:**
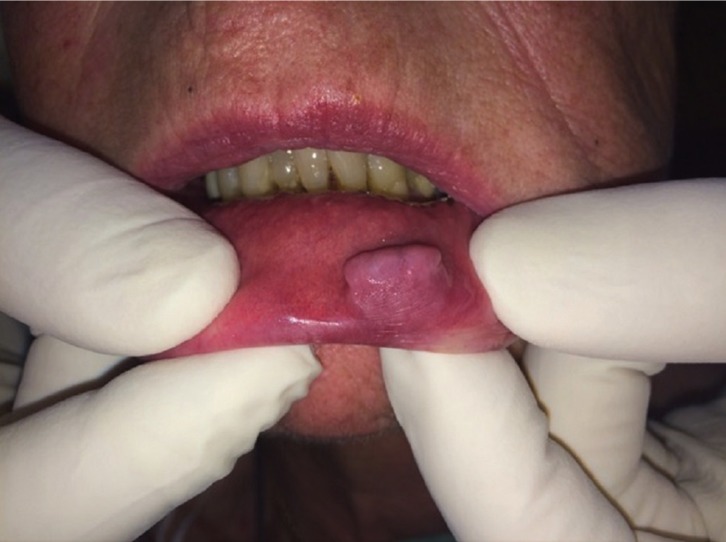
Exophytic nodule on the lower lip in patient 1.

An 80-year-old, non-smoker, female patient presented to the outpatient Clinic of the Department of Oral Medicine and Oral Pathology, School of Dentistry, National and Kapodistrian University of Athens, Greece for evaluation of an asymptomatic, exophytic nodule located on the lower lip. The patient had first noticed the lesion 4 months ago and since then it has been invariable. Her medical history was unremarkable and the last blood tests where within normal limits. On clinical examination, a well – circumscribed, penduculated, painless soft nodule was noted on the left side of the lower lip (Fig. 1). The lesion was covered by normal mucosa and measured approximately 1.5 x 1cm. The remaining oral mucosa was normal. With a provisional diagnosis of a mucocele, the lesion was excised under local anesthesia and sent for histopathological examination.

**Figure 2:**
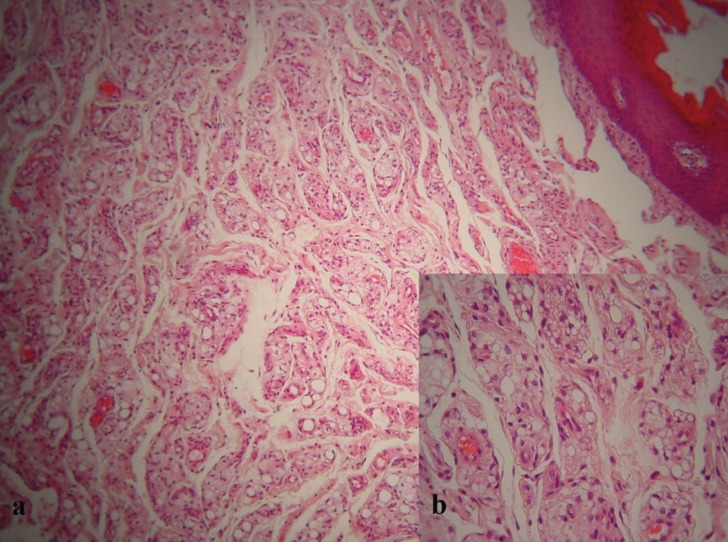
Multiple clear cystic spaces of varying size intermixed with epithelioid macrophages with vacuolated cystoplasm in a fibrous connective tissue stroma are observed. (a. hematoxylin-eosin stain, original magnification x100, b. hematoxylin-eosin stain, original magnification x200).

Microscopic examination of 5-mm-thick, formalin-fixed, paraffin-embedded tissue sections stained with hematoxylin-eosin revealed numerous, variably sized clear cystic spaces, some of which contained amorphous eosinophilic material, interspersed in a fibrous connective tissue stroma. These spaces were surrounded by vacuolated epithelioid histiocytes with a signet-ring like appearance intermixed with few multinucleated giant cells (Fig. 2a, 2b). Although the lesion was reminiscent of liposarcoma, no nuclear atypia was noticed and a foreign body granulomatous reaction was speculated; however polarized light microscopy did not reveal any birefringent material. Before setting a final diagnosis, the patient was contacted and asked if she had been subjected to any aesthetic procedure in the perioral area in the past. At that time, she recalled that she had received hyaluronic acid injections in both nasolabial folds and the lower lip 1.5 year before, however the histopathological features were compatible with silicone, a different filler from the one she thought she had received. The combination of the patient’s history and the microscopic examination set the final diagnosis of a FBG to silicone. Nine months after the excisional biopsy, no recurrence has been reported.

Case 2

**Figure 3:**
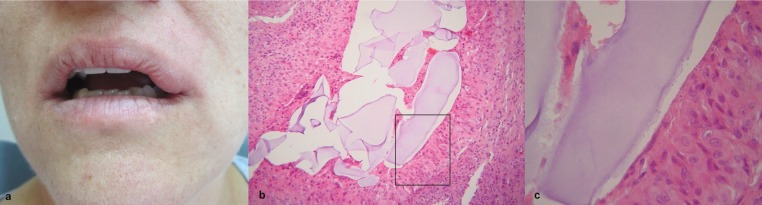
a. Extraoral view of the submucosal nodule on the upper left labial mucosa in patient 2. A pool of amorphous basophilic material corresponding to hyaluronic acid lined by epithelioid macrophages is observed (b. hematoxylin-eosin stain, original magnification x100, c. hematoxylin-eosin stain, original magnification x200).

A 48-year-old, non-smoker, female patient presented to a private oral medicine clinic with an asymptomatic, well-defined submucosal firm nodule on the upper lip, of 4 months duration. Her medical history was non – contributory while she had been subjected to hyaluronic acid filler injections in the ipsilateral nasolabial fold six months before her visit to the clinic. On clinical examination, a well - circumscribed submucosal nodule measuring approximately 2 x 1 cm was noticed on the left upper lip mucosa. The lesion was covered by normal-appearing mucosa and was painless and soft on palpation (Fig. 3a). The remaining oral mucosa was within normal limits. With a provisional diagnosis of foreign body granuloma and a differential diagnosis of salivary gland and neural tumor, the nodule was excised with no reported recurrence 4 months later. Histopathologically, pools of amorphous, basophilic material compatible with hyaluronic acid that were surrounded by epithelioid macrophages and mild lymphocytic inflammation were observed (Figs. 3b, 3c), confirming the tentative diagnosis of a FBG to hyaluronic acid filler.

## Discussion

The increasing demand for cosmetic procedures in the orofacial area, results in a growing number of complications, including the development of foreign body granulomas (FBGs). FBGs are histopathologically characterized by an organized collection of epithelioid histiocytes, containing foreign body material surrounded by chronic lymphocytic inflammation and fibrosis (6,12). A number of patients and practitioners accept this type of inflammatory lesions as normal as long as there is cosmetic improvement, whereas, from pathologists’ perspective, an inflammatory response to foreign material cannot be considered as normal tissue (12).

In addition to the present study, review of the pertinent English literature revealed 104 previously published cases of oral FBGs induced by STFs(1,3,6,9,11,13-37). The clinical and demographic data of all reported cases are summarized in  Table 2,  Table 2 continue. In all cases, diagnosis of FBG formation was rendered after histopathological examination. The mean age of the patients was 53,8±13,6 years (1,3,6,9,11,13,15,16,18-35) while the vast majority (98,1%) were women (1,3,6,9,11,13-37). This strong female predilection obviously reflects the fact that women prefer cosmetic procedures (6,37). The lower lip was the most common location of the lesions (30,5%) (1,3,6,22,24,31,33-36) followed by the upper lip (23,38%) (1,6,9,13,14,16,18,26,30,32). Most of the cases were clinically described as single, usually submucosal nodules (3,6,13,14,16,19,22,24,26,30) or masses (1,3,6,22,23,32,34). Concomitant redness or ulceration were scarcely reported whereas the majority of the patients (79%) reported no symptoms (6,11,13,14,16,19,21,26,28,29). The differential diagnosis varied depending on the clinical presentation and the location of the lesions. The literature to date shows that in cases which presented as single nodules or masses on the lips the differential diagnosis included mucoceles, benign salivary gland and soft tissue neoplasms, whereas diffuse lip swellings, were differentiated from orofacial granulomatosis, angioedema, Crohn’s disease and other less common oral conditions (6). The clinician should suspect a FBG if a patient reports a previous STF injection. Such a reaction may manifest many months or years after the injection (mean 2,9±4.2 years) (1,6,9,11,13-19,23-26,29,32) and may even develop in an area distant from the initial injection site (6). However, the diagnosis of a FBG may be challenging for both the clinician and the pathologist when a positive history of STF injection is not disclosed either because there is a long time lapse between the cosmetic procedure and the appearance of the lesion (3), or because the patients purposely conceal the cosmetic procedure for their own reasons(6).

**Table 2:**
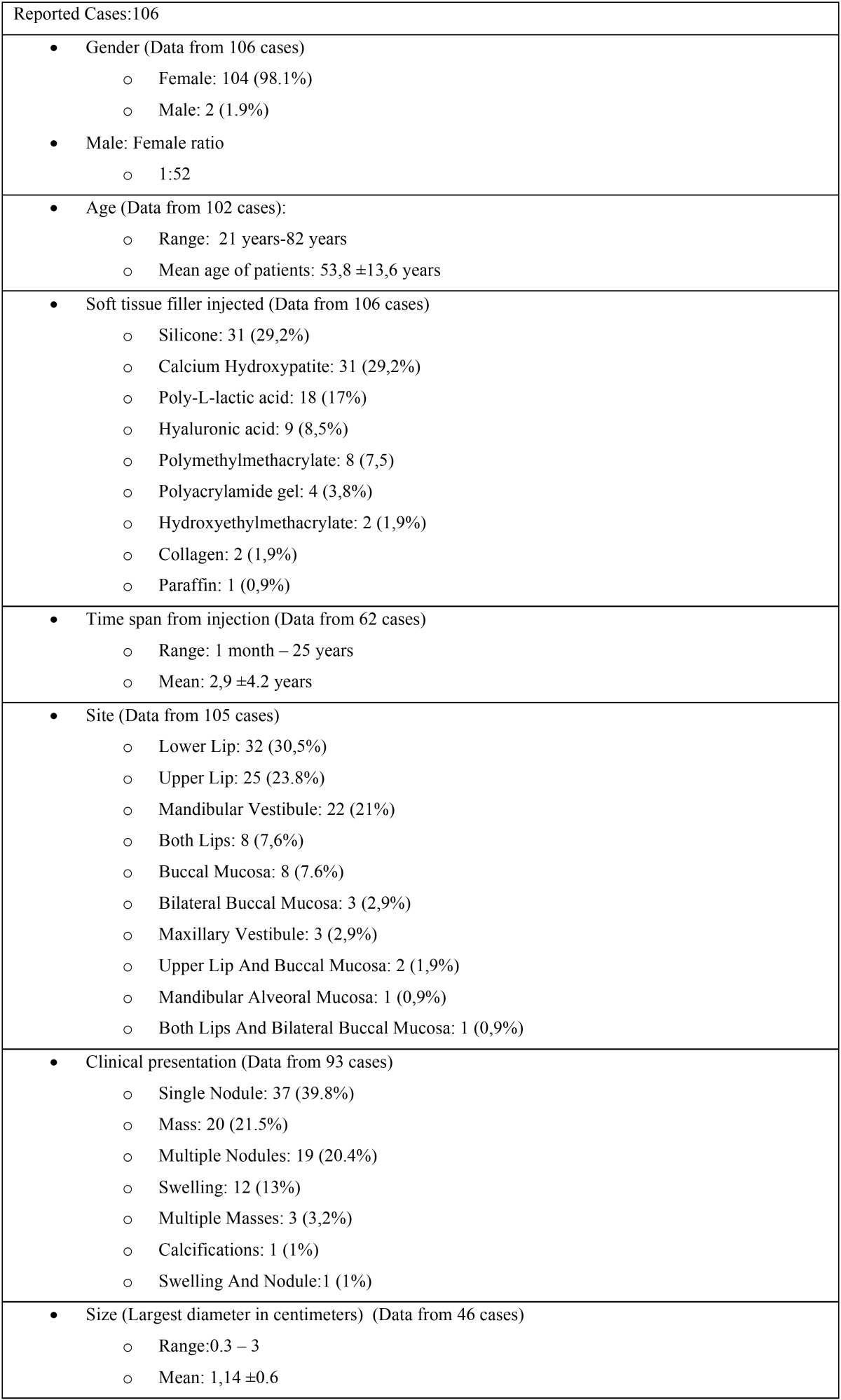
Clinical and demographic data of 104 previously published cases of oral foreign body granulomas induced by injectable soft tissue fillers, plus the 2 cases of the present study.

**Table 2 continue:**
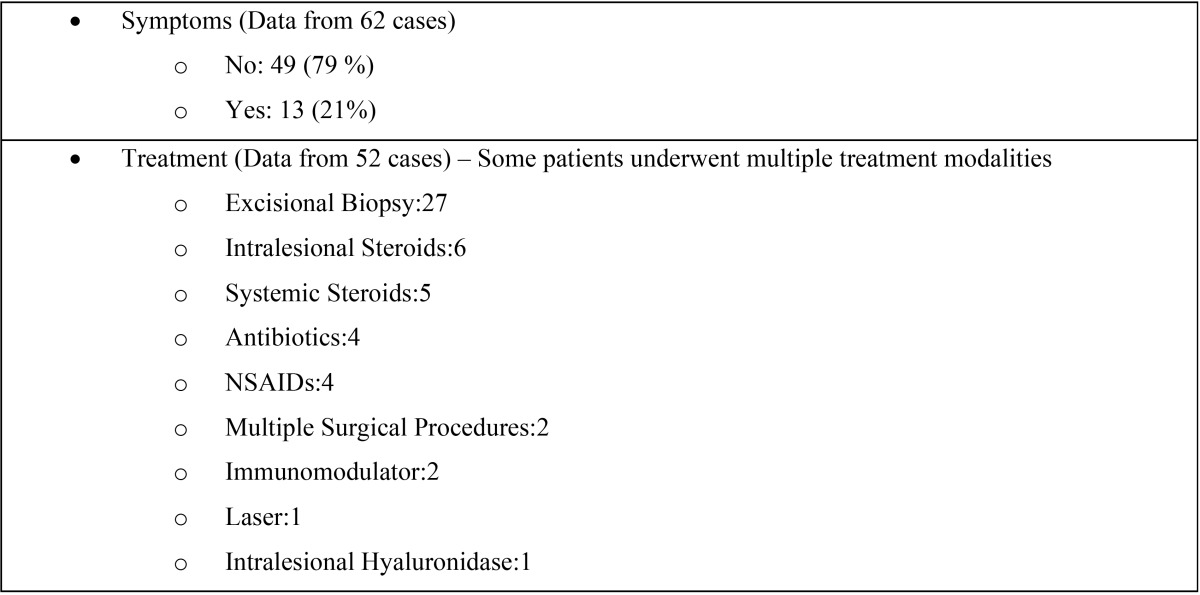
Clinical and demographic data of 104 previously published cases of oral foreign body granulomas induced by injectable soft tissue fillers, plus the 2 cases of the present study.

The responsible materials in most cases of FBG were silicone (3,6,14-16,21,22,27,35,36) and calcium hydroxyapatite (1,3,20,22,26,33,34), each one accounting for 29,2% of cases. FBGs induced by hyaluronic acid (3,6,18,22,31)and collagen (32,37),which are normal components of human and animal tissues, were scarce (8,5% and 1,9% respectively). In these cases, FBG formation was most probably due to cross-linking and increased concentration (2).

The diagnosis of a granulomatous foreign body reaction is rendered only after microscopic examination. In a single case report, fine needle aspiration cytology was reported as a less invasive method for diagnosis. However, histologic examination was also performed (23). The microscopic identification of the STF type is challenging if a detailed clinical history is not provided, in cases in which more than one filler is injected (37), or if the patient is not aware of the type of material used (3). Rarely, patients report a different type of STF from the one identified histopathologically, probably because the person who performed the injection withheld the true nature of the filler (6) as was the case in the first patient of the present study (case 1).

The histopathological appearance of granulomatous foreign body reactions on hematoxylin and eosin stain seem to be specific for each type of injected filler (3). Therefore, pathologists should be familiar with the characteristic microscopic features of each filler and not necessarily rely on the clinical history (12). Occasionally, two different fillers may be recognized in the same tissue sample, reflecting the injection of two types of fillers in the same patient (26). The histopathological patterns of FBG induced by the different types of STFs are presented in  Table 3.

**Table 3:**
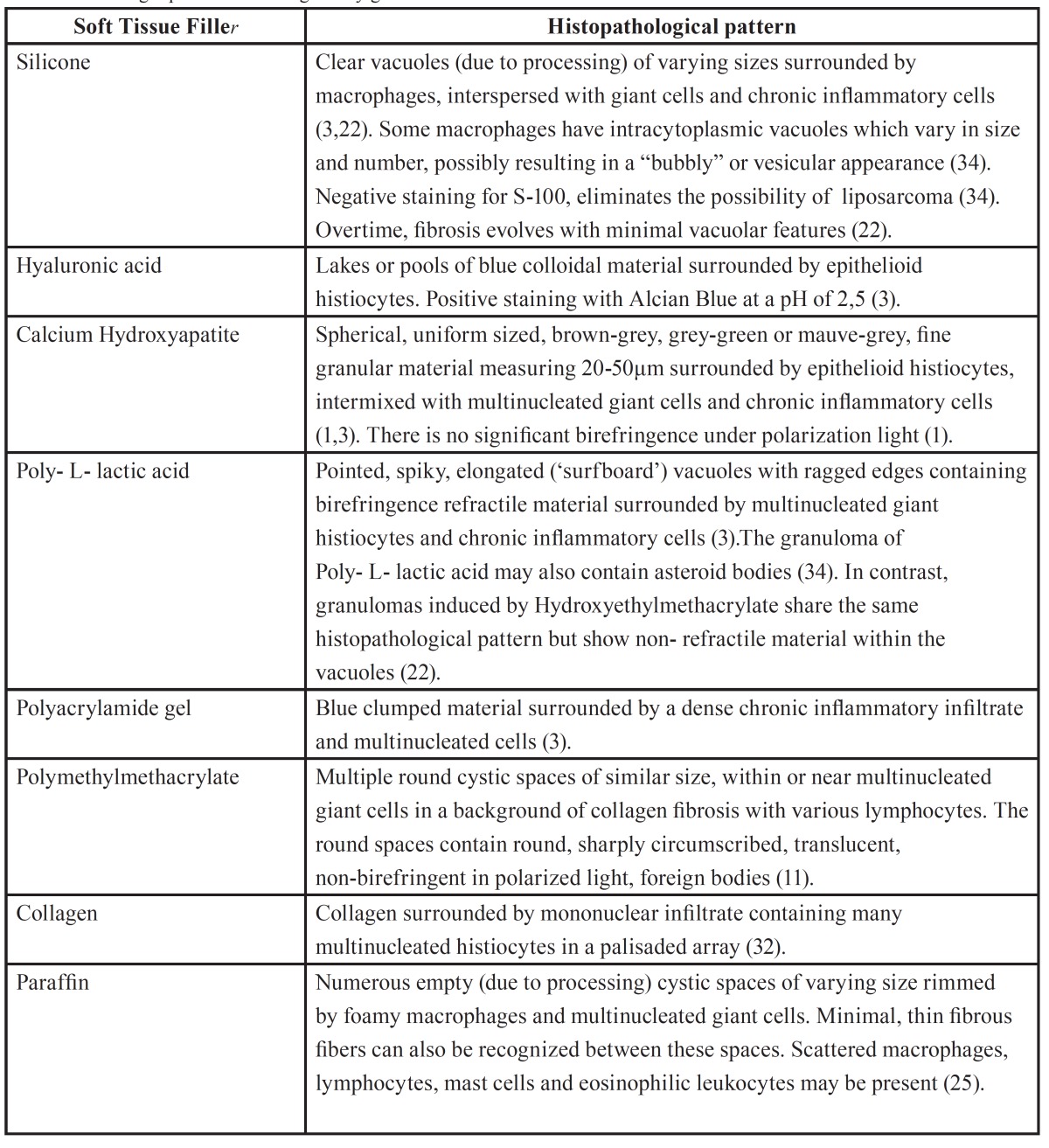
Histologic patterns of foreign body granulomas to different soft tissue fillers.

Many factors have been implicated in the etiology of FBGs to STFs such as the volume of the injected filler, STF impurities, the surface and the size of the particles (38) or a delayed hypersensitivity reaction (39). However, increasing evidence indicates that granuloma formation may be related to bacterial biofilms (8,10) which adhere to the surface of the filler when the procedure is not performed properly (9,39,40). The bacteria, which are organized in biofilms, are protected from the host immune system, thus remaining in a stable condition (9,39). Sometime later, ranging from months to years, they may be activated either by another filler injection, dental surgery, infection, trauma or unknown causes (2,39) leading to a host inflammatory response in the form of a granulomatous foreign body reaction(9,39). Polymerase Chain Reaction and pyrosequencing techniques may also be of help to identify the responsible bacteria (9).

Several different treatment approaches have been used (6,9,11,13,15-21,23-25,28,29) as seen in  Table 2. Intralesional steroids represent the first line of treatment; sparing systemic steroids for recurrent lesions, in doses higher than those used before locally (38). The role of antibiotics in FBG treatment is debatable (2). Excision of FBGs should be reserved as the last option, especially when they appear clinically as multiple nodules or as a diffuse swelling, in which case it may be impossible to remove the entire injected material (10,38). However, when FBG presents clinically as a single nodule, as in both of our cases, excisional biopsy is both diagnostic and therapeutic.

## Conclusions

• Although rare, foreign body granulomas to soft tissue fillers may develop months to several years after the injection. Due to the increasing use of soft tissue fillers, during history taking, the clinician should include questions regarding esthetic procedures performed in the perioral area.

• The pathologist should be familiar with the histologic pattern of each type of filler and not rely solely on the patient’s history.

• The treatment of choice for foreign body granulomas is prevention. Clinicians must be highly educated and trained to perform the injections under aseptic conditions, thus minimizing the risk of infection.
